# Non-Thermal Atmospheric Pressure Plasma Efficiently Promotes the Proliferation of Adipose Tissue-Derived Stem Cells by Activating NO-Response Pathways

**DOI:** 10.1038/srep39298

**Published:** 2016-12-19

**Authors:** Jeongyeon Park, Hyunyoung Lee, Hae June Lee, Gyoo Cheon Kim, Do Young Kim, Sungbum Han, Kiwon Song

**Affiliations:** 1 Department of Biochemistry, College of Life Science and Biotechnology, Yonsei University, Seoul 03722, Korea; 2 Department of Electrical Engineering, Pusan National University, Pusan 46241, Korea; 3 Department of Oral Anatomy, School of Dentistry, Pusan National University, Yangsan 50612, Korea; 4 Department of Dermatology and Cutaneous Biology Research Institute, Yonsei University College of Medicine, Seoul 03722, Korea; 5 Batang Plastic Surgery Center, Gangnam-Gu, Seoul 06120, Korea

## Abstract

Non-thermal atmospheric pressure plasma (NTAPP) is defined as a partially ionized gas with electrically charged particles at atmospheric pressure. Our study showed that exposure to NTAPP generated in a helium-based dielectric barrier discharge (DBD) device increased the proliferation of adipose tissue-derived stem cells (ASCs) by 1.57-fold on an average, compared with untreated cells at 72 h after initial NTAPP exposure. NTAPP-exposed ASCs maintained their stemness, capability to differentiate into adipocytes but did not show cellular senescence. Therefore, we suggested that NTAPP can be used to increase the proliferation of ASCs without affecting their stem cell properties. When ASCs were exposed to NTAPP in the presence of a nitric oxide (NO) scavenger, the proliferation-enhancing effect of NTAPP was not obvious. Meanwhile, the proliferation of NTAPP-exposed ASCs was not much changed in the presence of scavengers for reactive oxygen species (ROS). Also, Akt, ERK1/2, and NF-κB were activated in ASCs after NTAPP exposure. These results demonstrated that NO rather than ROS is responsible for the enhanced proliferation of ASCs following NTAPP exposure. Taken together, this study suggests that NTAPP would be an efficient tool for use in the medical application of ASCs both *in vitro* and *in vivo*.

Plasma is described as a quasi-neutral mixture of charged particles and radicals in a partially ionized gas. Recently, many studies attempted to take advantage of the low temperature of non-thermal atmospheric pressure plasmas (NTAPPs) for biomedical applications owing to the controllability of plasma chemistry and kinetics (for reviews, see Fridman *et al*.^1^, Kong *et al*.^2^, and Lee *et al*.^3^). NTAPPs are easily generated in air and can be used without causing thermal damage to cells. Effects of NTAPPs on living tissues include sterilization, wound healing, and changes in cell migration (for a review, see Park *et al*.^4^). The different effects of plasma depend on plasma dosage and their complex chemical compositions. Recently, the clinical applications of NTAPPs have become a very active research area.

Previous studies regarding the clinical application of NTAPP with respect to human cells have focused on its ability to induce necrosis^5^ or apoptosis^6^,^7^,^8^. Several research groups have demonstrated that NTAPP induces apoptosis in cancer cells^9^,^10^ and reduces tumor size in mouse xenograft models *in vivo*^11^, thereby suggesting the use of NTAPP in cancer therapy (for a review, see Song *et al*.^12^). Increasing evidence suggests that reactive oxygen species (ROS) are the major players in NTAPP-induced apoptosis *in vitro*^13^,^14^,^15^. However, there is a discrepancy between the cytotoxic effect of non-thermal plasma and ozone, which is a considerable component of non-thermal air plasma^16^,^17^.

In our previous study, we showed that NTAPP exposure selectively induces apoptosis in cancer cells by activating the ROS response system; however, it accelerated the proliferation of normal fibroblast IMR 90 cells and adipose tissue-derived stem cells (ASCs)^18^. NTAPP has also been reported to accelerate wound healing processes by activating the nuclear factor erythroid-related factor 2 (NRF2) signaling pathway in human keratinocyte HaCa T cell line *in vitro*^19^, and to promote re-epithelialization and wound closure by activating keratinocytes and fibroblasts in Wistar rats’ wound skin^20^. These studies strongly suggested that NTAPP stimulates the proliferation of normal and adult stem cells.

ASCs are mesenchymal stem cells (MSC) that have the potential to differentiate into various cell types such as adipocytes, osteoblasts, chondrocytes, and neurons^21^. ASCs are also capable for self-renewal, which is an important property of stem cells to regenerate damaged tissues^22^. ASCs are relatively easy to isolate from adipose tissues by liposuction and may provide an accessible source of adult stem cells for use in regenerative medicine (for reviews, Bunnell *et al*.^23^, and Mizuno *et al*.^24^). However, in general, it is difficult to culture adult stem cells *in vitro* while ensuring that they maintain their stemness; moreover, adult stem cells undergo rapid senescence *in vitro*^25^,^26^,^27^.

Biomarkers expressed on the cell surface are generally used to identify adult stem cells. For ASCs, CD44 and CD105 are used as positive markers, while CD45 and FABP4 are used as negative markers. CD44 is a well-accepted stem cell marker^28^,^29^,^30^,^31^, while CD105 is mainly expressed in human mesenchymal stem cells including ASCs isolated from adipose tissue^22^,^30^,^31^,^32^. CD45 is a pan-leukocyte marker that is well-expressed on hematopoietic stem cells but not on ASCs^29^,^30^,^32^,^33^,^34^,^35^. Fatty acid binding protein 4 (FABP4) is a specific maker found on ASCs that have differentiated into adipocytes^36^.

In this study, we focused on the effect of NTAPP on ASCs and its mechanisms. We showed that NTAPP can enhance the proliferation of ASCs *in vitro*, thereby supporting the potential applications of NTAPP in the field of regenerative medicine.

## Results

### Design of a helium-based dielectric barrier discharge (DBD) type NTAPP device

**Figure 1:**
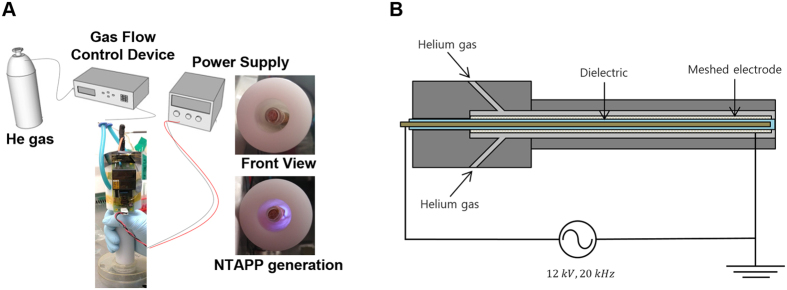
Helium-based dielectric barrier discharge type device used for non-thermal atmospheric pressure plasma (NTAPP) generation. (**A**) Schematic description of the NTAPP-generating device used in this study (photographed by J. Park). (**B**) Inner components of the device that generate NTAPP (drawn by H. Lee).

The schematics of the experimental setup are shown in Fig. 1. The dielectric barrier discharge (DBD)-type atmospheric pressure plasma device is connected to an alternating current (AC) voltage supply and a gas feeding system, as shown in Fig. 1A. The DBD device is composed of a grounded cylindrical meshed electrode, a dielectric glass tube with a diameter of 6.35 mm, and a concentric electrode rod located inside the glass tube, as shown in Fig. 1B. A Teflon body forms a gas flow tube with an inner diameter of 14 mm. The device was designed to be fed with two types of gas through two inlets; however, only helium (He) gas was applied in the current experiment. The flow rate of the feeding gases was controlled between 1~10 slm by a mass flow controller. The peak-to-peak sinusoidal voltage was applied to the central rod from 0 to 12 kV at 20 kHz, while the meshed electrode was grounded. Thus, a surface discharge was generated between the cylindrical glass and the mesh covering it. The direction of the electric field is perpendicular to the direction of gas flow, and reactive species rather than charged particles are ejected through the gas outlet. This is the main difference between this device and a conventional plasma jet^37^,^38^,^39^ that delivers charged particles as well as radicals. This device generates a large amount of helium atoms in the excited state in the discharge region inside the long tube, which is very effective for the generation of reactive nitrogen species (RNS) and reactive oxygen species (ROS) by the Penning effect outside.

### NTAPP accelerates the proliferation of ASCs but induces apoptosis in HeLa cells

**Figure 2:**
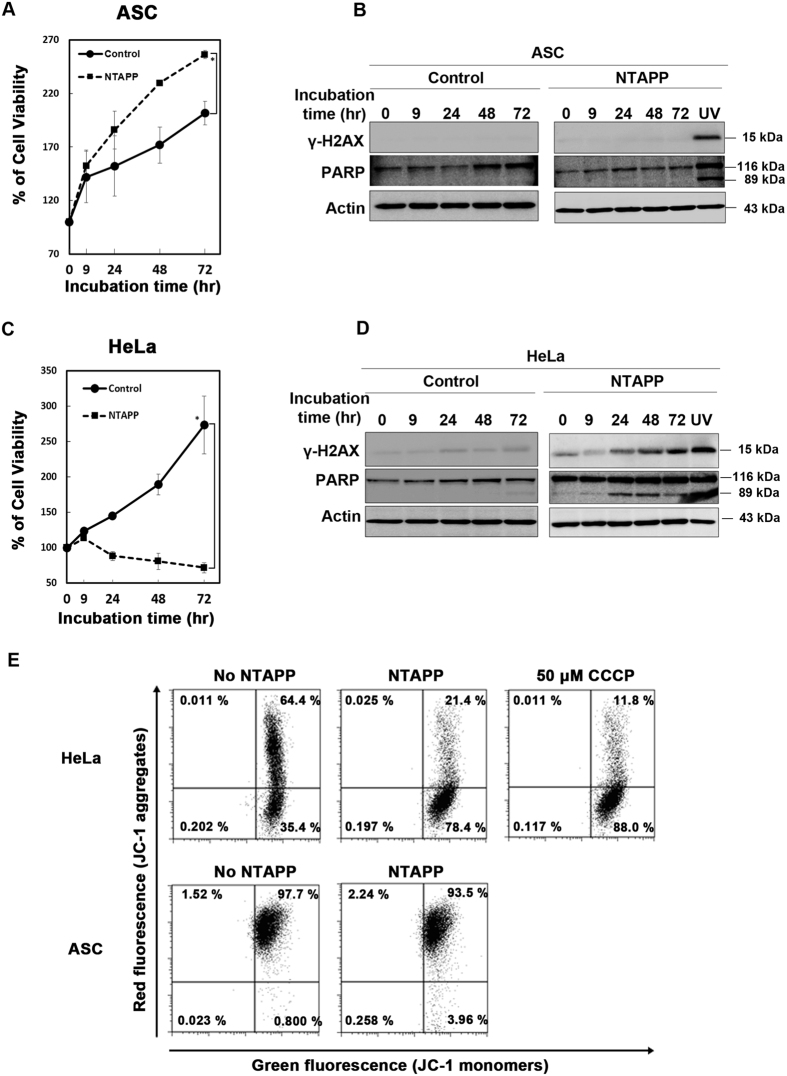
Non-thermal atmospheric pressure plasma (NTAPP) accelerates the proliferation of adipose tissue-derived stem cells (ASCs) but induces apoptosis in HeLa cells. (**A–D**) ASCs (**A,B,E**) and HeLa cells (**C,D,E**) were exposed to NTAPP for a total of 10 times, for 50 sec every h, and were further incubated for 72 h from the initial exposure. Cell viability was evaluated at each indicated incubation time-point. (**A,C**) Cell viability was measured by MTT assay, and all results were represented as mean ± SD. N = 4. P < 0.05 (*) indicates significant differences compared with the control. (**B,D**) Western blot analysis of ASCs (**B**) and HeLa cells (**D**) were performed to assess the expression of γ-H2AX and PARP following NTAPP exposure. Actin was used as the loading control. Cells exposed to UV were used as the positive control for DNA damage and cell death. (**E**) The mitochondrial membrane potential was monitored in NTAPP-treated HeLa and ASCs. Cells were stained with 2 μM JC-1 dye for 30 min at 37 °C, and both red and green fluorescence emissions were analyzed by flow cytometry. Cells treated with 50 μM carbonyl cyanide 3-chlorophenylhydrazone (CCCP) for 4 h prior to JC-1 staining were used as a positive control for mitochondrial membrane disruption.

Our previous study demonstrated that NTAPP selectively induces apoptosis in various cancer cells, but increased the proliferation of normal fibroblast IMR90 cells and ASCs^18^. Here, we examined whether NTAPP could promote the proliferation of ASCs by using helium-based DBD-type NTAPP. To compare the effect of NTAPP between adult stem cells and cancer cells, we exposed NTAPP to ASCs and HeLa cells for a total of 10 times, for 50 sec each time every hour, and further incubated the cells for 72 h after the initial NTAPP exposure. Viability of NTAPP-exposed ASCs increased 1.57-fold on an average, compared with that observed with the unexposed control cells, as determined by MTT assays at 72 h (Fig. 2A). However, the viability of NTAPP-exposed HeLa cells was significantly decreased compared to that of the unexposed control cells (Fig. 2C).

Because NTAPP induces apoptosis in various cancer cells through DNA damage^40^, we also tested whether NTAPP induces DNA damage and apoptosis in ASCs and HeLa cells. The expression of γ-H2AX (a marker for DNA double strand break), and the cleavage of caspase-3 and PARP (markers of apoptotic cells), were not detected in NTAPP-exposed ASCs (Fig. 2B, Supplementary Fig. S1A); however, we observed a time-dependent increase in the expression of γ-H2AX, and activation of caspase-3 and PARP by cleavage in HeLa cells exposed to NTAPP (Fig. 2D, Supplementary Fig. S1B). These results demonstrated that the same exposure of NTAPP accelerated the proliferation of ASCs but induced apoptosis in HeLa cells.

In order to further confirm that NTAPP induces apoptosis in HeLa cells but has no apoptotic effect in ASCs, we examined the depolarization of the mitochondrial membrane potential in NTAPP-treated ASCs and HeLa cells by using JC-1 dye (5′,6,6′-tetrachloro-1,1′,3,3′-tetraethylbenzimidazolylcarbocyanine iodide). It has been well documented that mitochondria membrane potential changes in the early stage of apoptosis, in which mitochondrial membrane permeability increases, leading to the release of the pro-apoptotic factor cytochrome c^6^,^41^. JC-1 is a mitochondrial membrane-specific fluorescent dye that measures changes in mitochondrial membrane potential (for a review, see Cottet-Rousselle *et al*.^42^). JC-1 can form J-aggregates (red fluorescence at 585 nm) in the mitochondrial inner membrane in normal conditions with the mitochondria membrane intact, but it cannot be transported into the mitochondria when mitochondrial membrane potential is destructed and is present as monomers (green fluorescence at 530 nm) at the cytoplasm^6^,^8^,^43^,^44^. JC-1 reversibly changes its fluorescence color from green to red as the mitochondria membrane becomes polarized^45^,^46^. Also, JC-1 dye-especially J-aggregates with red fluorescence- responds linearly to mitochondrial membrane potential changes, and the red/green ratio and mitochondrial membrane potential values are highly correlated^44^,^46^,^47^. In normal conditions with usual mitochondrial membrane potentials, multiple regions with red and green fluorescence are observed because not all mitochondria in the same cell sustain the same mitochondrial membrane potential^43^,^44^,^48^. As a positive control for JC-1, we first treated HeLa cells with 50 μM CCCP (carbonyl cyanide 3-chlorophenylhydrazone) that has been known to disrupt mitochondrial membrane potential^49^,^50^. By CCCP treatment, the ratio of HeLa cells with red-negative and green-positive fluorescence increased to 88.0% (Fig. 2E, upper panel). When we exposed NTAPP to HeLa cells for 10 times and incubated for a total of 72 h, the ratio of cells with red-negative and green-positive fluorescence (apoptotic cells) increased to 78.4%, strongly demonstrating the depolarization of mitochondrial membranes in apoptosis by NTAPP treatment. On the other hand, in the NTAPP-unexposed control HeLa cells, the ratio of cells with JC-1 red-negative and green-positive fluorescence was 35.4% (Fig. 2E, upper panel).

When we applied JC-1 to both NTAPP-treated ASCs and the untreated control, very similar high ratios of both red and green-positive fluorescence were detected in both NTAPP-treated and –untreated ASCs (Fig. 2E, lower panel), demonstrating that NTAPP did not induce the depolarization of mitochondrial membrane potential in ASCs. These observations strongly supported that NTAPP- exposed ASCs neither change their mitochondrial membrane potential nor undergo apoptosis. These results also confirmed that exposure to NTAPP induces apoptosis in HeLa cells but not in ASCs.

### NTAPP-exposed ASCs maintain their stemness

**Figure 3:**
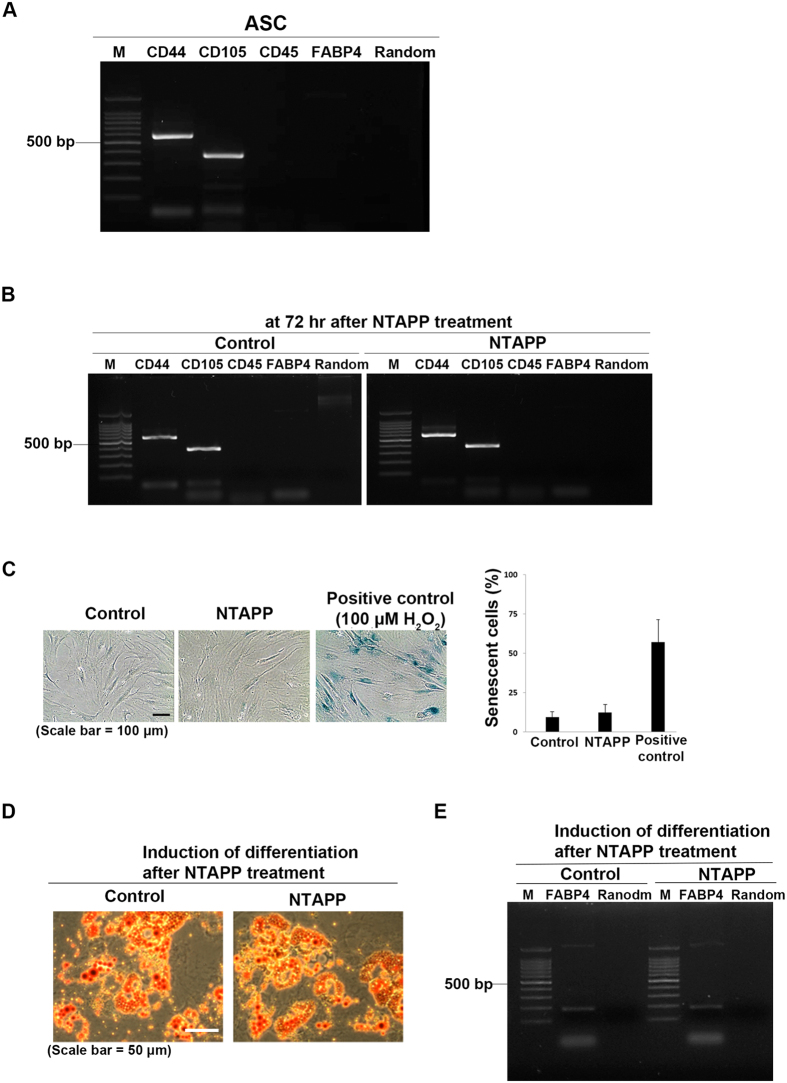
Non-thermal atmospheric pressure plasma (NTAPP)-exposed adipose tissue-derived stem cells (ASCs) maintain their stem cell properties. (**A**) Reverse transcription-polymerase chain reaction (RT-PCR) was performed by using RNA extracted from ASCs. CD44 and CD105 were used as positive markers and CD45 was used as a negative marker for the analysis of ASCs. FABP4 was used as a differentiation marker of ASCs. (**B**) Expression of the markers of ASCs was analyzed by RT-PCR at 72 h after the first NTAPP exposure and compared to that in unexposed control cells. (**C**) SA-βGal assay was performed to evaluate senescence in ASCs at 72 h after exposure to NTAPP for a total of 10 times. ASCs treated with 100 μM H_2_O_2_ were used as the positive control. Scale bar, 100 μm. Senescent cells were counted, and the values were expressed as percentages. (**D**) Differentiation of NTAPP-exposed ASCs into adipocytes was induced by incubation for 28 days in adipogenic differentiation medium, and the differentiation of the ASCs was detected using Oil-red O staining. Scale bar, 50 μm. (**E**) ASCs were evaluated for the expression of an adipocyte marker, FABP4, by using RT-PCR. Random primers were used as negative controls for RT-PCRs.

In order to use NTAPP to accelerate the proliferation of ASCs for different applications, the characteristic of ASCs must be maintained after NTAPP exposure. We compared the stemness characteristics of NTAPP-exposed and -unexposed ASCs. CD44 and CD105 were used as positive markers, CD45 was used as a negative marker, and FABP4 was used as a differentiation marker to evaluate the characteristics of ASCs^30^,^36^. Before NTAPP exposure, we confirmed the expression of these markers in ASCs by using reverse transcription-polymerase chain reaction (RT-PCR). CD44 and CD105 were expressed, while CD45 and FABP4 were not detected in ASCs, as shown in Fig. 3A. We then exposed the ASCs to NTAPP for a total of 10 times, and incubated the cells for 72 h after the first exposure. At the indicated time, we monitored the expression of the markers and observed that CD44 and CD105 continued to be expressed, while CD45 and FABP4 were not expressed, identical to that observed in NTAPP-unexposed ASCs (Fig. 3B).

The expression of these markers was further confirmed in NTAPP-exposed ASCs using flow cytometric analysis with anti-CD44-phycoerythrin (PE), anti-CD105-allophycocyanin (APC), and anti-CD45-fluorescein isothiocyanate (FITC). As shown in Supplementary Fig. S2A, the NTAPP-treated ASCs at 72 h incubation after the initial NTAPP exposure showed high expression of both CD44 and CD105 but no expression of CD45, similar to that observed in NTAPP-unexposed ASCs. These results further support that NTAPP exposure does not change the stemness characteristics of ASCs.

It has been reported that most stem cells including human mesenchymal stem cells (hMSCs) are prone to genotoxic damages that eventually lead to cellular senescence when cells proliferate *in vitro*^51^,^52^,^53^. Thus, we monitored whether ASCs showing increased proliferation following NTAPP exposure underwent cellular senescence by using senescence-associated β-galactosidase staining. Cells treated with 100 μM H_2_O_2_ were used as the positive control for cell senescence. As shown in Fig. 3C, only 12% of NTAPP-exposed ASCs were positive for β-galactosidase staining, similar to that observed with unexposed ASCs (9%), while 57% of H_2_O_2_-treated positive control cells were positive for β-galactosidase staining. These results suggest that NTAPP did not cause cellular senescence in ASCs while it promoted the proliferation of ASCs.

ASCs can be differentiated into several cell types such as adipocytes, neurons, osteoblasts, and chondrocytes (for a review, see Locke *et al*.^54^). To confirm that ASCs showing increased proliferation following NTAPP exposure can maintain their capability to differentiate into various cell types, we examined the differentiation of NTAPP-exposed ASCs into adipocytes. ASCs that were exposed to NTAPP for a total of 10 times for 50 s each time every hour, were incubated for 72 h, and subsequently incubated in adipogenic differentiation medium for 28 days. Cells that differentiated into adipocytes were analyzed using Oil-red O staining for the visualization of intracellular lipids, and their RNA samples were subjected to RT-PCR to evaluate the expression of FABP4. Cells exposed to NTAPP formed intracellular lipids at a level similar to that observed in unexposed cells (Fig. 3D). In addition, both unexposed and NTAPP-exposed cells expressed FABP4 after differentiation (Fig. 3E). In order to compare the differentiation efficiency of NTAPP-exposed ASCs with the unexposed control, we also counted the number of differentiated ASCs in NTAPP-exposed and -unexposed cells after induction of differentiation into adipocytes. As shown in Supplementary Fig. S2B, the percentage of differentiated cells in NTAPP-exposed ASCs (73.28%) was similar with that of unexposed control cells (71.40%). These results demonstrated that NTAPP-exposed ASCs showing increased proliferation continued to maintain their ability to differentiate. Collectively, these observations strongly suggest that NTAPP accelerates the proliferation of ASCs without inducing cell senescence and that NTAPP-exposed ASCs retain their stemness characteristics and their ability to differentiate.

### NO plays a major role in NTAPP-induced proliferation of ASCs

**Figure 4:**
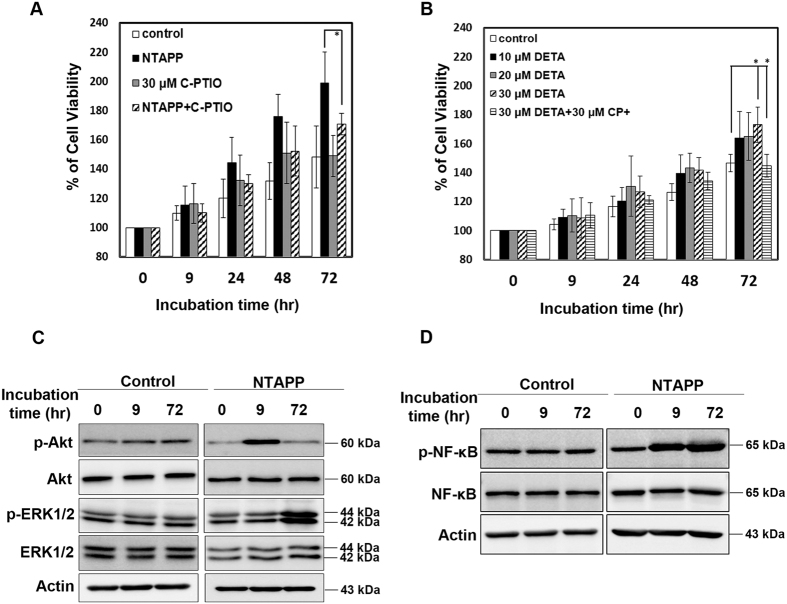
NO plays a key role in non-thermal atmospheric pressure plasma (NTAPP)-induced proliferation of adipose tissue-derived stem cells. (**A**) ASCs pretreated with culture medium alone (as the negative control) or 30 μM carboxy-PTIO were exposed to NTAPP for a total of 10 times. Cells were totally incubated for 72 h after the initial NTAPP exposure. Cell viability was measured by MTT assays, and the results were represented as mean ± SEM; N = 4. P < 0.05 (*) indicates significant differences among samples. (**B**) Different concentrations of DETA-NONOate (untreated, 10, 20, and 30 μM) were added to the medium containing ASCs. carboxy-PTIO (30 μM) was added to medium containing 30 μM DETA. Cell viability was evaluated by MTT assay, and the results were represented as mean ± SEM. N = 4. P < 0.05 (*) indicates significant differences compared with each sample. (**C,D**) The expression of (**C**) Akt, phospho-Akt, ERK1/2, and phospho-ERK1/2, and (**D**) NF-κB and phospho-NF-κB in NTAPP-exposed ASCs was analyzed by western blot at 0, 9, and 72 h from the initial exposure. Actin was used as the loading control.

Nitric Oxide (NO) is a well-known second messenger and a key modulator in many physiological functions including cell proliferation (for a review, see Villalobo *et al*.^55^). NTAPP generates ROS and RNS; among these species, plasma can easily generate NO from N_2_ and O_2_ in the air. Given that NO at a low concentration has been reported to promote cell proliferation through the inhibition of cellular apoptosis (for a review, see Napoli *et al*.^56^) and NTAPP exposure is known to promote proliferation in ASCs, we hypothesized that NO might play a role in enhancing the proliferation of ASCs following NTAPP exposure. To examine whether NO generated by NTAPP affects the proliferation of ASCs, we treated the cells with carboxy-PTIO, a NO scavenger, with or without NTAPP exposure. Viability was analyzed after the cells were exposed to NTAPP (control cells were not exposed) in the presence or absence of a NO scavenger in the medium. The viability of NTAPP-exposed cells increased by 199% at 72 h after NTAPP exposure, compared with that at the beginning of incubation (0 h; considered 100%), while the viability of unexposed cells increased only by 148% at 72 h. However, the viability of NTAPP-exposed cells following treatment with carboxy-PTIO was reduced to 170% (Fig. 4A). These observations revealed that NO is mainly responsible for the increased proliferation of NTAPP-exposed ASCs.

We then tested whether NO itself can activate the proliferation of ASCs. ASCs were treated with different concentrations (10, 20, and 30 μM) of an NO donor, DETA-NONOate^57^,^58^, and incubated for 9, 24, 48, and 72 h. Following treatment with the NO donor, the viability of ASCs increased in a dose-dependent manner to a greater extent than that observed with untreated cells. To verify the enhanced proliferation of ASCs by NO, we co-treated the ASCs with 30 μM carboxy-PTIO and the same concentration of the NO donor, and examined whether the NO scavenger can compensate for the effect of the NO donor. When ASCs were co-treated with both the NO scavenger and the NO donor, viability was found to be similar to that of untreated cells (Fig. 4B), suggesting that NO, at a particular concentration, promotes the proliferation of ASCs.

To further verify that NO is responsible for the enhanced proliferation of ASCs following NTAPP exposure, we investigated the related cellular pathways of NO-induced cell proliferation. NO is known to be produced by activated nitric oxide synthase (NOS) via the PI-3K/Akt signaling pathway^59^,^60^ and to induce the mitogen-activated protein kinase (MAPK)/ERK pathway that leads to cell proliferation^61^,^62^. Also, it has been well reported that both PI-3K/Akt and MAPK/ERK signaling pathways activate the phosphorylation of NF-κB and allow it to enter the nucleus, thereby promoting cell proliferation by NF-κB-dependent transcription^63^,^64^. Thus, we examined the activation of Akt, ERK1/2, and NF-κB at 0, 9, and 72 h after the exposure of ASCs to NTAPP. The expression of phospho-Akt was increased in ASCs immediately after NTAPP exposure, which was administered 10 times, but decreased to the normal level at 72 h after the initial NTAPP exposure. Phospho-ERK1/2 was elevated at 72 h (Fig. 4C). These results demonstrated that NTAPP promoted the proliferation of ASCs by activating the Akt and ERK signaling pathways at different time-points. We further confirmed the activation of Akt and ERK signaling pathways in the NTAPP-treated ASCs by monitoring the phosphorylation of NF-κB that is activated by Akt and ERK. Compared with the untreated control, NF-κB phosphorylation was highly increased in NTAPP-treated ASCs at 9 h and 72 h after the initial NTAPP exposure (Fig. 4D). Taken together, these observations demonstrated that NTAPP promotes the proliferation of ASCs via NO by activating Akt, ERK1/2, and their downstream NF-κB.

### ROS are not involved in NTAPP-induced proliferation of ASCs

**Figure 5:**
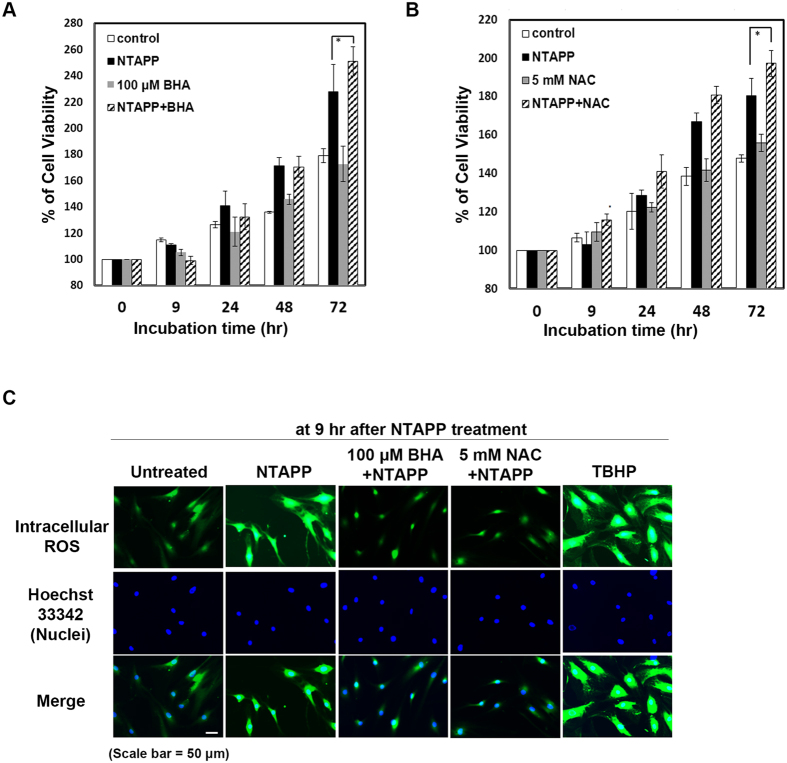
Reactive oxygen species (ROS) are not responsible for non-thermal atmospheric pressure plasma (NTAPP)-induced proliferation of ASCs. (**A,B**) ASCs were pretreated with culture medium alone, 100 μM butylated hydroxyl anisole (BHA; **A**), or 5 mM N-acetylcysteine (NAC; **B**) and exposed to NTAPP for a total of 10 times. Cells were further incubated for 72 h from the initial NTAPP exposure. The percentage of cell viability was measured by MTT assay, and the results were represented as mean ± SD. N = 4. P < 0.05 (*) indicate differences among each sample. (**C**) Untreated ASCs and those pretreated with 100 μM BHA or 5 mM NAC were exposed to NTAPP for a total of 10 times, and their intracellular ROS levels were monitored at 9 h from the initial exposure. Cells treated with 100 μM *tert*-butyl hydroperoxide (TBHP) were used as the positive control for ROS generation. Nuclei were stained with Hoechst 33342. Scale bar, 50 μm.

Many research groups have proposed that the diverse biomedical effects of NTAPP rely on the various ROS generated by NTAPP. Thus, we examined whether the increased proliferation of ASCs following NTAPP exposure is attributed to the ROS generated by NTAPP. For this purpose, we examined the viability of ASCs with and without NTAPP exposure in the presence or absence of anti-oxidants. We used butylated hydroxyanisole (BHA) as a free radical scavenger^65^ and N-acetylcysteine (NAC) as a thiol oxidant^66^. The viability of ASCs was significantly increased at 72 h after NTAPP treatment compared to that of untreated ASCs, as shown in Fig. 2A. If ROS were responsible for the proliferation of ASCs by NTAPP treatment, the cell viability would have decreased in the presence of anti-oxidants following NTAPP exposure. However, when the ASCs were exposed to NTAPP in the presence of anti-oxidants, viability was not reduced (Fig. 5A,B), suggesting that ROS do not play a role in the increased proliferation of ASCs.

We also monitored the level of intracellular ROS in ASCs after NTAPP exposure for a total of 10 times (9 h), using a fluorogenic marker of ROS, carboxy-H_2_DCFDA. Cells treated with *tert*-butyl hydroperoxide (TBHP) were used as the positive control for intracellular ROS generation^67^. ASCs accumulated intracellular ROS when exposed to NTAPP for 50 sec every h for a total of 10 times, compared with that observed with the untreated cells. In the presence of an anti-oxidant (100 μM BHA or 5 mM NAC), the intracellular ROS levels were efficiently reduced in NTAPP-exposed ASCs, although their increased proliferation was not affected (Fig. 5C). These results indicated that intracellular ROS generated in ASCs following NTAPP exposure are not responsible for the increased proliferation of ASCs following NTAPP exposure.

## Discussion

In recent years, NTAPP has been studied for its clinical applications, especially in cancer therapy and sterilization^38^,^68^,^69^. While NTAPP has been known to induce apoptosis in various cancer cells^70^,^71^, its role in the activation of proliferation is not well investigated. In this study, we used a helium-based dielectric barrier discharge (DBD)-type NTAPP device generating multiple intracellular ROS/RNS but not much ozone^18^, demonstrating that NTAPP promotes the proliferation of ASCs, while maintaining the stem cell characteristics of ASCs. We also showed that nitric oxide (NO) generated from NTAPP plays a key role in NTAPP-induced increased proliferation of ASCs by activating the Akt and ERK1/2 pathways. Collectively, these results strongly suggest that NTAPP can increase the efficiency of ASC culture *in vitro*, thereby supporting the potential applications of NTAPP in the field of regenerative medicine.

NO acts as an intracellular messenger and regulator in biological functions such as immune responses, apoptosis, cell proliferation, and angiogenesis (for reviews, see Villalobo *et al*.^55^, Bogdan *et al*.^72^, Brune *et al*.^73^, and Morbidelli *et al*.^74^). NO has been known to be biosynthesized endogenously by various nitric oxide synthase (NOS) enzymes activated through the PI3K-Akt signaling pathway^59^. NO acts through the stimulation of soluble guanylate cyclase (sGC) to form cyclic-GMP (cGMP), which activates protein kinase G (PKG), leading to the activation of the ERK signaling pathway for cell proliferation^61^,^75^,^76^. Interestingly, different cell fates depend on NO concentrations: low NO concentration promotes cell survival and proliferation in various cells including stem cells^77^, while high NO concentration leads to cell cycle arrest and cell death^78^. NO is generated by NTAPP. Our study showed that NO generated by NTAPP plays an important role in inducing the proliferation of ASCs. However, not only NO but also other unknown factors might be involved in the increased proliferation of ASCs following NTAPP exposure because the viability of ASCs following combined treatment with NTAPP and NO scavenger was not recovered to the level in control cells even though viability was reduced, as shown in Fig. 4A. Furthermore, when ASCs were treated with an NO donor, DETA-NONOate, cell proliferation increased but not to the extent observed in NTAPP-exposed ASCs. Further studies would be necessary to understand which other components of NTAPP are responsible for the promotion of NTAPP-induced cell proliferation.

As expected from the results in Fig. 4 that show that the increased proliferation of ASCs was mainly attributed to NO, we observed the activation of Akt and ERK1/2 in NTAPP-exposed ASCs. However, their time of activation was different (Fig. 4C). A study of the mechanism underlying the differential regulation of the Akt and ERK signaling pathways would be necessary to understand the mechanism by which NO controls the proliferation of adult stem cells including ASCs.

ROS generated by NTAPP may be responsible for the diverse biomedical effects of NTAPP^6^,^15^,^79^. ROS have also been reported to induce various biological effects and determine cell fate in stem cells and cancer cells, depending on their concentrations. At low levels, ROS actively promote cell proliferation, migration, and differentiation (for a review, see Maraldi *et al*.^80^). ROS also help stem cells maintain their stemness (for a review, see Chaudhari *et al*.^81^). In contrast, high concentrations of ROS induce cell senescence and cell death (for reviews, see Wang *et al*.^82^, and Liou *et al*.^83^). Our results showed that ROS generated by He-based NTAPP was not responsible for the increased proliferation of ASCs and they did not induce senescence or apoptosis in ASCs.

The results of this study show the potential of NTAPP to be used to control the proliferation of ASCs and suggest a clue as to why NTAPP activates wound healing in tissues. To develop NTAPP as a reliable tool for use in stem cell technology and regeneration, the effect of NTAPP on other stem cells needs to be investigated and further chemical evaluations of NTAPP will be necessary.

## Materials and Methods

### Isolation of ASCs from adipose tissue and culture

Adipose tissue was obtained during elective surgeries with informed consent of patient, and all experiments involving adipose tissue were performed in accordance with the guidelines approved by the Severance Hospital Institutional Review Board (IRB No. 4-2014-0830). ASCs were isolated from the tissue as described previously by Ma *et al*.^18^. Briefly, adipose tissue was repeatedly washed with PBS, and 0.0075% collagenase (Sigma-Aldrich, MO, USA) was added to the tissue sample at 37 °C with shaking for 1 h. The sample was centrifuged for 10 min at 1000 rpm to remove the top layer of oil, fat, and the layer of collagenase solution to obtain the stromal vascular fraction (SVF) pellet. The pelleted SVF was suspended in 155 mM NH_4_Cl to lyse red blood cells at room temperature for 10 min. ASCs were then collected by centrifugation at 1000 rpm for 10 min.

Human adipose tissue-derived stem cells (ASCs) were maintained in Dulbecco’s modified Eagle’s medium (DMEM)/Ham’s F-12 supplemented with 10% (v/v) fetal bovine serum (FBS; Sigma-Aldrich, MO, USA) and 10 ml/l penicillin-streptomycin (GIBCO, NY, USA). HeLa cells were maintained and grown in DMEM containing 10% FBS and 10 ml/l penicillin-streptomycin. All cells were maintained at 37 °C in an atmosphere containing 5% CO_2_.

### NTAPP exposure and cell viability assay

To expose cells to NTAPP, 3 × 10^4^ cells seeded in 35-mm culture dishes were incubated for 24 h. Cells were exposed to the indicated dose (5 standard liter/min, 20 V) of NTAPP for 50 sec every h for a total of 10 times, and the NTAPP-exposed cells were further incubated for 63 h (a total of 72 h after the first NTAPP exposure). The distance between the device and cells was fixed to 1 cm, and 1.5 ml of medium was used.

After exposure to NTAPP, cell viability was measured by adding 1 ml of 5% 3-(4,5-dimethylthiazol-2-yl)-2,5-diphenyltetrazolium bromide (MTT; Amresco Inc., OH, USA) to cells in a dish, which was followed by incubation of the cells for 1.5-3 h at 37 °C in an atmosphere containing 5% CO_2_. The formazan produced was dissolved in 1 ml dimethyl sulfoxide (DMSO) and measured using an ELISA reader (SoftMax Pro 4.0, Molecular Devices) at 570 nm^84^.

### Reverse transcription-polymerase chain reaction

ASCs were harvested and their total RNA was extracted using the RNeasy Mini kit (Cat. No. 74101, QIAGEN, Germany). Using purified total RNA, cDNA was synthesized using the Biotechnology Power cDNA synthesis kit (Cat. No. 25011, iNtRON, Korea), and PCR was performed using the synthesized cDNA along with primers specific for CD44, CD45, CD105, and FABP4, using the *i*-MAX DNA polymerase kit (Cat. No. 25234, iNtRON, Korea). PCR products were detected by electrophoresis on a 2% agarose gel. The primers used for PCR were shown in the Table 1.

### Characterization of ASC membrane antigens by flow cytometry

ASCs were trypsinized at 72 h after exposure to NTAPP for a total of 10 times, washed, and centrifuged for 10 min at 1000 rpm. Cells were incubated with the optimal dilution of fluorescein- conjugated monoclonal antibodies (mAbs) for 30 min on ice: anti-CD44-phycoerythrin (PE; eBioscience, CA, USA), anti-CD105-allophycocyanin (APC; eBioscience, CA, USA), and anti-CD45-fluorescein isothiocyanate (FITC; eBioscience, CA, USA). 10,000 cells per assay were counted using a FACSCalibur flow cytometer (BD Bioscience, CA, USA) and analyzed by FlowJo software (FlowJo LLC, OR, USA).

### Analysis of mitochondrial membrane potential (MMP) with JC-1

Cells were harvested at 72 h after the initial exposure to NTAPP, and stained with 2 μM JC-1 dye for 30 min at 37 °C by following the instructions of MitoProbe JC-1 assay kit (Thermo Fisher Scientific, OR, USA). As a positive control of membrane potential disruption, cells were also treated with 50 μM carbonyl cyanide 3-chlorophenylhydrazone (CCCP) for 4 h prior to JC-1 staining. Flow cytometry was used to evaluate JC-1 fluorescence in both HeLa cells and ASCs. Data were analyzed using FlowJo software (FlowJo LLC, OR, USA).

### Western blot analysis

NTAPP-exposed cells were harvested and lysed as described previously^18^. Histones were extracted with 0.5 N HCl and neutralized with 1 M NaOH. Total protein samples (40 μg) or histones were separated by SDS-PAGE and detected using the following primary antibodies: anti-poly ADP-ribose polymerase (PARP; Cell Signaling Technology, Inc., MA, USA), anti-phospho-H2AX (γ-H2AX; Millipore, Germany), anti-caspase-3 (Cell Signaling Technology, Inc., MA, USA), anti-actin (Cell Signaling Technology, Inc., MA, USA), anti-ERK1/2 (Cell Signaling Technology, Inc., MA, USA), anti-phospho-ERK1/2 (Cell Signaling Technology, Inc., MA, USA), anti-Akt (Cell Signaling Technology, Inc., MA, USA), anti-phospho-Akt (Cell Signaling Technology, Inc., MA, USA), anti-NF-κB (Cell Signaling Technology, Inc., MA, USA), and anti-phospho- NF-κB (Cell Signaling Technology, Inc., MA, USA). An enhanced chemiluminescence system (Amersham Biosciences) was used for the blot analysis.

### Treatment of ASCs with the NO donor and NO scavenger

Cells were treated with 10, 20, or 30 μM DETA-NONOate (Cayman Chemical Company, MI, USA) to generate NO and cell proliferation was monitored by the MTT assays. Cells were pretreated with 30 μM carboxy-PTIO {[(2-(4-carboxyphenyl)-4,4,5,5-tetramethylimidazoline-1-oxyl-3-oxide)]; Sigma-Aldrich, MO, USA}, a NO scavenger, prior to NTAPP exposure.

### Detection of intracellular ROS

Cells were treated with 5 mM NAC (Sigma-Aldrich, MO, USA) and 100 μM BHA (Sigma-Aldrich, MO, USA), intracellular ROS scavengers, prior to NTAPP exposure. TBHP was used to generate intracellular ROS as a positive control. Intracellular ROS were measured using the ROS detection kit (Invitrogen, CA, USA), following the manufacturer’s protocol. Cells were observed by fluorescence microscopy on an Axioplan2 (Zeiss) under 200× objective.

### Senescence-associated (SA) β-galactosidase activity assay

Cells exposed to NTAPP for a total of 10 times were incubated for 72 h and further incubated for 4 days after replacing the medium. Cells treated with 100 μM H_2_O_2_ were used as a positive control for senescence. Next, the cells were fixed in 2% formaldehyde and 0.2% glutaraldehyde for 15 min at room temperature and monitored by senescence-associated β-galactosidase (SA-βGal) staining with X-gal (Sigma-Aldrich, MO, USA), as described previously^85^. Cells were evaluated using an OLYMPUS CKX41 microscope under a 100× objective.

### Differentiation into adipocytes and Oil-red O staining

ASCs were induced to differentiate into adipocytes by incubating them in the adipogenic differentiation medium (200 μM indomethacin, 0.5 mM IBMX, 1 μM dexamethasone, and 10 μM insulin in DMEM/F12 medium supplemented with 10% fetal bovine serum and 1% penicillin-streptomycin) for 28 days as reported previously^86^. To determine lipid accumulation, adipocytes were fixed with 4% para-formaldehyde for 10 min and stained with Oil red O solution for 15 min as described previously^87^. Cells were evaluated using the OLYMPUS CKX41 microscope under a 200× objective.

### Statistical analysis

Statistical analysis was performed using GraphPad Prism 6 (GraphPad Software, Inc., CA, USA). Data are represented with the mean ± standard deviation (S.D.) (Figs 2A,C and 5A,B) of at least three repeated experiments or with the standard error of the mean (S.E.M.) (Fig. 4A,B) of more than three independent experiments. We applied non-parametric Mann-Whitney U test to assess statistically significant differences^88^. P < 0.05 (*) indicates statistical significance compared with the control.

## Additional Information

**How to cite this article:** Park, J. *et al*. Non-Thermal Atmospheric Pressure Plasma Efficiently Promotes the Proliferation of Adipose Tissue-Derived Stem Cells by Activating NO-Response Pathways. *Sci. Rep.* **6**, 39298; doi: 10.1038/srep39298 (2016).

**Publisher's note:** Springer Nature remains neutral with regard to jurisdictional claims in published maps and institutional affiliations.

## Supplementary Material

Supplementary Figures

## Figures and Tables

**Table 1:** The primers used to detect cell surface markers by PCR.

Cell surface marker	Oligonucleotide primer used
CD44	5′-GATCCACCCCAACTCATCT-3′ (forward)
	5′-AACTGCAAGAATCAAAGCCA-3′ (reverse)
CD105	5′-TGTCTCACTTCATGCCTCCAGCT-3′ (forward)
	5′-AGGCTGTCCATGTTGAGGCAGT-3′ (reverse)
CD45	5′-ACCAGGGGTTGAAAAGTTTCAG-3′ (forward)
	5′-GGGATTCCAGGTAATTACTCC-3′ (reverse)
FABP4	5′-ACTGGGCCAGGAATTTGACG-3′ (forward)
	5′-CTCGTGGAAGTGACGCCTT-3′ (reverse)
