# Supplementary material for: Non-Thermal Atmospheric Pressure Plasma Efficiently Promotes the Proliferation of Adipose Tissue-Derived Stem Cells by Activating NO-Response Pathways
Source: Sci Rep. 2016 Dec 19;6:39298. doi: 10.1038/srep39298 (PMC5171835; doi:10.1038/srep39298)

< Supplementary information >

Non-Thermal Atmospheric Pressure Plasma Efficiently Promotes the Proliferation of Adipose Tissue-Derived Stem Cells by Activating NO-Response Pathways

Jeongyeon Park[1], Hyunyoung Lee[2], Hae June Lee[2], Gyoo Cheon Kim[3], Do Young Kim[4], Sungbum Han[5], and Kiwon Song[1,]**

[1]Department of Biochemistry, College of Life Science and Biotechnology, Yonsei University, Seoul 03722, Korea

[2]Department of Electrical Engineering, Pusan National University, Pusan 46241, Korea

[3]Department of Oral Anatomy, School of Dentistry, Pusan National University, Yangsan 50612, Korea

[4]Department of Dermatology and Cutaneous Biology Research Institute, Yonsei University College of Medicine, Seoul 03722, Korea

[5]JW Plastic Surgery Center, Seoul 06025, Korea

**All correspondence should be addressed to Kiwon Song

Department of Biochemistry, College of Life Science and Biotechnology, Yonsei University, Seoul 03722, Korea

Tel: 82-2-2123-2705; Fax: 82-2-362-9897; E-mail: bc5012@yonsei.ac.kr

Legends to figures

Supplementary Figure S1. Non-thermal atmospheric pressure plasma (NTAPP) induces the activation of caspase-3 in HeLa cells but not in adipose tissue-derived stem cells (ASCs). (A, B) In NTAPP-exposed ASCs (A) and HeLa cells (B), the expression of caspase-3 and cleaved caspase-3 was analyzed by western blots. Actin was used as a loading control. Cells treated to UV were used as a positive control for cell death.

Supplementary Figure S2. Non-thermal atmospheric pressure plasma (NTAPP)-exposed adipose tissue-derived stem cells (ASCs) maintain their stemness characteristics. (A) Flow cytometry was performed to analyze the expression of surface markers, CD44, CD105, and CD45, in the NTAPP-exposed ASCs at 72 h after the initial exposure and in NTAPP-untreated control ASCs. (B) The NTAPP-exposed and -unexposed ASCs were induced to differentiate into adipocytes in adipogenic differentiation medium for 28 days, and the number of adipocytes was counted after Oil red O staining. The relative percentage of differentiated adipocytes was plotted with standard deviations.

Supplementary Figure S1

A

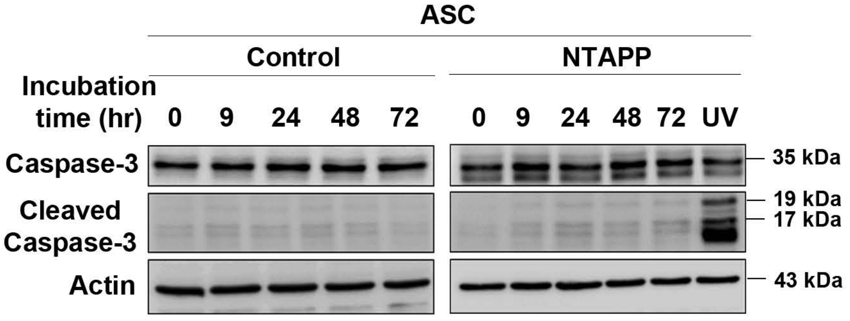

B

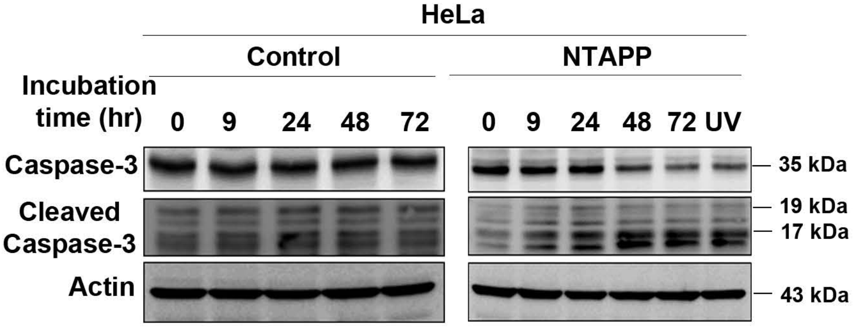

Supplementary Figure S2

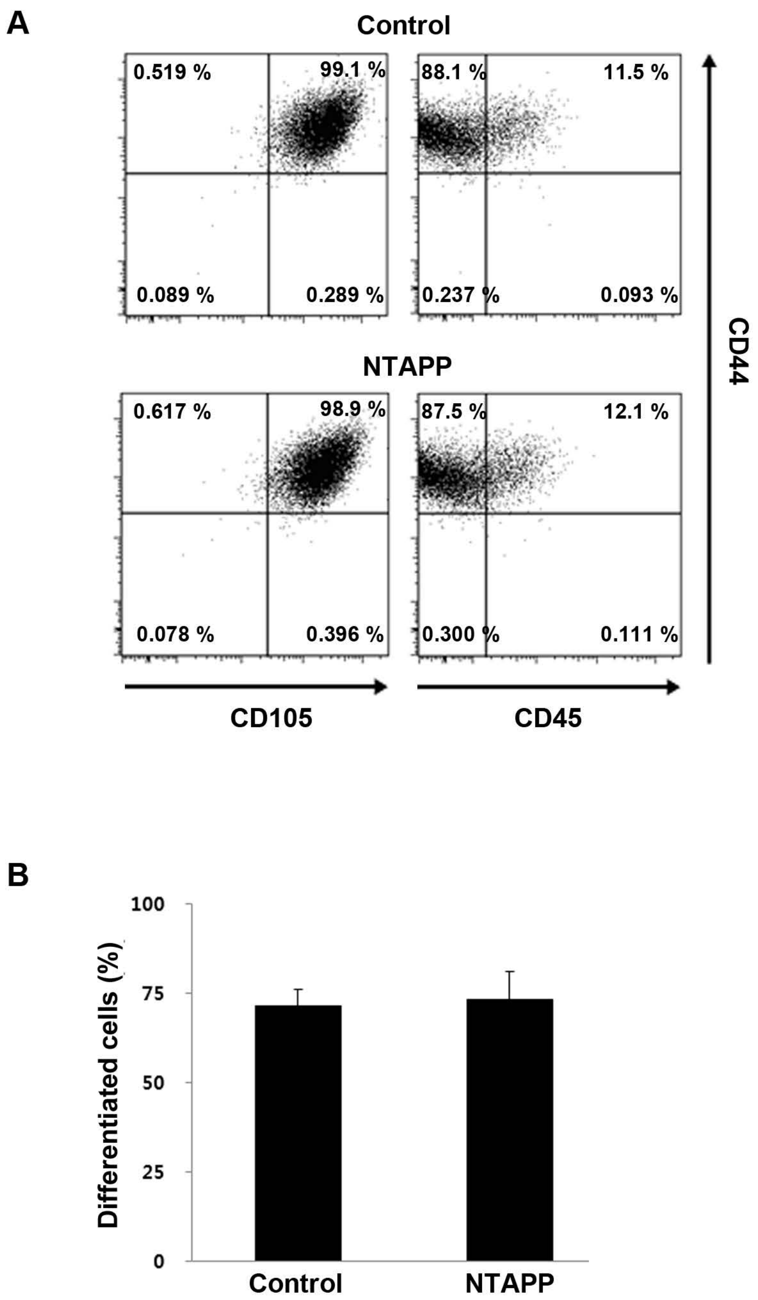

Supplement: Supplementary Figures [file srep39298-s1.pdf]
